# Beyond “pains” and “gains”: untangling the health consequences of probation

**DOI:** 10.1186/s40352-022-00193-7

**Published:** 2022-10-01

**Authors:** Michelle S. Phelps, Ingie H. Osman, Christopher E. Robertson, Rebecca J. Shlafer

**Affiliations:** 1 Department of Sociology, University of Minnesota, 909 Social Sciences Building, 267 19th Ave. S, Minneapolis, MN 55455 USA; 2 Department of Pediatrics, University of Minnesota, Minneapolis, MN USA

**Keywords:** Probation, Community supervision, Substance-related disorders, Mental health, Inequality

## Abstract

**Background:**

Research on the health consequences of criminal legal system contact has increasingly looked beyond imprisonment to understand how more routine forms of surveillance and punishment shape wellbeing. One of these sites is probation, the largest form of supervision in the U.S. Drawing on an interview study with 162 adults on probation in Hennepin County, MN, in 2019, we map how adults on probation understand the consequences of supervision for their health and how these self-reported health changes correlate with individual, social, and structural circumstances.

**Results:**

Roughly half of participants described their health as having improved since starting probation, while the remainder were split between no change and worsened health. Examining both closed-ended survey questions and open-ended interview prompts, we find that the “gains” of supervision were correlated with substance use treatment (often mandated), reduced drug and alcohol use, increased housing and food security, and perceptions of support from their probation officer. However, these potentially health-promoting mechanisms were attenuated for many participants by the significant “pains” of supervision, including the threat of revocation, which sometimes impacted mental health. In addition, participants in the most precarious circumstances were often unable to meet the demands of supervision, resulting in further punishment.

**Conclusions:**

Moving beyond the “pains” and “gains” framework, we argue that this analysis provides empirical evidence for the importance of moving social services outside of punishing criminal legal system interventions. People with criminal legal contact often come from deeply marginalized socio-economic contexts and are then expected to meet the rigorous demands of supervision with little state aid for redressing structural barriers. Access to essential services, including healthcare, food, and housing, without the threat of further criminal legal sanctions, can better prevent and respond to many of the behaviors that are currently criminalized in the U.S. legal system, including substance use.

## Background

Over the past two decades, scholars, policymakers, and the public have become increasingly aware of the harms of mass incarceration. Confinement in penal facilities is associated with a host of long-term impacts, including housing, employment, and family difficulties (Kirk & Wakefield, 2018), which together imperil health and wellbeing for individuals, their families, and communities (Blankenship et al., 2018; Massoglia & Pridemore, 2015). As research on the health-related consequences of mass incarceration burgeons, scholars have begun to look beyond the prison to examine how other stages of the criminal legal system—from police contact to arrest, court processing, jail stays, and community supervision—impact health (Fernandes, 2020). A key site in this carceral archipelago is probation, the most common form of correctional control in the United States (U.S.).

By the close of 2020, 3.9 million adults in the U.S. were on probation, compared to the 862,100 on parole (Kaeble, 2021) and 1.2 million in the nation’s state and federal prisons (Carson, 2021). For the majority of adults who are on “active” supervision,^1^ probation typically entails supervision by a probation officer (PO) who monitors compliance with a number of conditions (e.g., abstain from alcohol and drug use, report regularly, complete mandatory programs, and pay legal financial obligations). If persons violate these conditions, they can be revoked to jail or prison, either for a short term of incarceration or the full suspended sentence (Doherty, 2016; Hayes, 2018; Klingele, 2013; McNeill, 2019; Phelps & Ruhland, 2022). Probation supervision rates, like other forms of criminal legal system involvement, are highest among the urban poor, especially in Black, Indigenous, and Latina/o/x communities, social contexts that both increase the likelihood of legal contact and morbidity and mortality risks (Fernandes, 2020; Semenza & Link, 2019). As a result, adults on probation experience elevated rates of chronic mental and physical health conditions, including substance use disorders (Fearn et al., 2016; Han et al., 2020; Hawks et al., 2020; Vaughn et al., 2012; Winkelman et al., 2020); higher age-adjusted mortality rates (Wildeman et al., 2019); and lower rates of health insurance (Knapp et al., 2019) than the general population.

Despite the burgeoning interest in estimating the health of adults on probation, little qualitative research to-date has asked people to describe the impact of probation on their health and wellbeing. Previous qualitative research on the experiences of people on supervision suggests that probation can serve as an intervention that reduces risky behaviors and helps connect adults to necessary services (the “gains” of supervision), but also imposes substantial “pains,” including material and psychological burdens (Phelps & Ruhland, 2022; Hayes, 2018; Huebner & Shannon, 2022; McNeill, 2019; Welsh, 2017). While this research suggests that probation may both ameliorate and exacerbate health conditions, previous qualitative work has not investigated how adults on probation themselves understand these impacts. In this paper, we use interviews with adults on probation to ask: (1) How do adults on probation report their health has changed since starting probation? (2) What are the individual, social, and structural factors associated with health changes while on probation?

### Mass probation and health in the U.S.

Begun in the Progressive Era as a rehabilitative alternative to incarceration, probation exists at the center of the “penal-welfare continuum” that governs the lives of the poor in the U.S. (Brydolf-Horwitz & Beckett, 2021). At its peak in 2007, 1 in every 53 adults was on probation compared to 1 in 198 in prison) (Phelps, 2017), with rates highest among young Black men without a high school diploma (Phelps, 2018). As probation grew, it also became increasingly neoliberal, piling on requirements, shifting the cost of supervision and burden of rehabilitation onto the people under supervision, and increasing the risk of revocation to jail or prison (Feeley & Simon, 1992; Phelps & Ruhland, 2022).

Probation typically requires abiding by a set of “conditions,” or requirements for living in the community, which, if violated, can lead to technical violations and potentially the revocation of the probation sentence to jail or prison. Conditions of probation often include reporting to one’s PO, securing formal employment and a permanent residence, maintaining sobriety, participating in mandatory substance use disorder (SUD) and mental health treatment programs, and avoiding contact with police and individuals with felonies (Corbett Jr., 2015; Doherty, 2016). To monitor compliance, POs may conduct job and home visits, administer drug tests, and collect fines and fees.

Yet in addition to surveillance, POs can provide social work case management services, including helping people access (sometimes through mandated participation) substance use treatment programs, mental health treatment, housing assistance, and employment resources (McNeill, 2019; Taxman, 2012). As Phelps and Ruhland (2022) summarize, probation thus provides a coercive form of care that provides streamlined access to life-sustaining resources while also burdening precarious adults with financial, emotional, and time demands. Or, as Hayes (2018) reviews, supervision entails both “pains” and “gains.” These benefits and costs of supervision, however, can vary dramatically across individuals, POs, offices, departments, states, and countries (Doherty, 2016; Petersilia, 2002; Huebner & Shannon, 2022).

As a central location of both punishment and aid, probation has significant public health consequences. Yet scholars have focused most of their attention on the health consequences of imprisonment rather than probation or community supervision. Several recent papers address this gap, using the National Survey of Drug Use and Health (NSDUH), a national survey of the household population in the U.S. These studies find that adults who report being on probation in the past year are significantly more likely to experience a higher burden of chronic health conditions, mental health conditions, physical and cognitive disabilities, comorbid health conditions, substance use disorders, and greater levels of risk for serious illness or death from COVID-19, compared to the age-adjusted general population (Vaughn et al., 2012; Fearn et al., 2016; Han et al., 2020; Hawks et al., 2020; Winkelman et al., 2020; Gutierrez & Patterson, 2021). Despite these needs, adults on probation are less likely to have insurance coverage compared to the general population (Hawks et al., 2020; Knapp et al., 2019) and less likely to receive appropriate outpatient care for their health conditions (Hawks et al., 2020; Olson et al., 2021). Vaughn et al. (2012) show, however, that adults on probation and parole in the past year were more likely than the general population to have accessed substance use treatment, though this elevated rate likely reflects differential treatment needs more than preferential access (Lorvick et al., 2015). Examining mortality rates, Wildeman et al. (2019) find that adults on probation face higher rates of mortality than both the general population and incarcerated adults.

Few studies have examined the *mechanisms* through which probation might improve or worsen individuals’ health and wellbeing (though see Lorvick et al., 2015). Previous qualitative research with adults on probation on the experience of supervision more broadly, however, suggests that probation may have multiple and contradictory effects on health. On one hand, a criminal record confers a stigmatized identity that can lower an individual’s subjective social status, which is an important predictor of health and driver of health disparities (Demakakos et al., 2008; Schnittker & Bacak, 2013). Likewise, having a criminal record introduces significant barriers to finding affordable, stable housing in safe neighborhoods (Bryan, 2022; Cobbina et al., 2014; Herbert et al., 2015; Rosenberg et al., 2021) and reliable, well-paying, and dignified employment (Huebner & Shannon, 2022; Pager, 2007). These barriers relegate individuals on probation to precarious, low-wage employment and hazardous occupational environments (Zatz, 2020) as well as chronic housing instability (Burgard et al., 2012), both key social determinants of health and correlates of revocation and future prison stays (Hamilton et al., 2015; Holtfreter et al., 2004). In addition, supervision itself may produce direct stressors, including a loss of autonomy, that worsen health (Thoits, 2010).

On the other side of the ledger, by requiring adults on probation to report verified home addresses, search for (or maintain) employment, and avoid drug and alcohol use, supervision may provide coercive motivation to ameliorate living conditions that improve health. Perhaps most importantly, case management services through probation can connect adults on probation to services, programs, and government assistance. Adopting the risk-need-responsivity (RNR) model, many community corrections departments use risk-needs assessment tools to assess the kinds of assistance that might be helpful in reducing the risk of recidivism (Andrews & Bonta, 2010). This facilitated and/or coerced provision of services may include access to state identification, benefits like public health insurance, and healthcare services, including substance use treatment. Indeed, as Brydolf-Horwitz and Beckett (2021) note, many of the substance use treatment beds available to low-income adults are funded through correctional agencies and reserved for justice-involved adults (see also Miller & Stuart, 2017). Increased access to substance use treatment programs can reduce the morbidity and mortality risks faced by justice-involved adults (He & Barkowski, 2020; Maclean & Saloner, 2019; Western & Simes, 2019), as well as reduce arrests, crime, and recidivism (Edwards et al., 2022; Galvin et al., 2021; Moore et al., 2020; Simes & Jahn, 2022). Other research, however, cautions that such programs often provide only short-term respite rather than longer-term stability, come with significant financial and time costs, and do not meet the multiple and overlapping health and support needs of people involved in the criminal legal system (Gowan & Whetstone, 2012; Halushka, 2020; Kerrison, 2019; Odio et al., 2018; Sered & Norton-Hawk, 2019).

Together, this work suggests that probation may have both ameliorative and corrosive consequences for adults’ health and wellbeing. It is also worth noting that these effects are likely bi-directional; just as probation may have complex impacts on health, so might individuals’ physical and mental wellbeing likely shape their ability to complete the demands of supervision (Thomas et al., 2015; Link et al., 2019). Our contribution is to look directly at adults’ experiences on probation to better understand the mechanisms linking supervision and health.

## Methods

Our study was focused on Hennepin County, Minnesota, which is the state’s largest county and includes the city of Minneapolis. While rates of imprisonment in Minnesota are relatively low, the state has one of the country’s highest community supervision rates (Phelps, 2017). We recruited participants for this study by posting flyers in probation offices, the drug testing center, and local health and social service agencies that serve justice-involved populations. Participants were also recruited through referrals from previous participants, though most learned about the study by seeing flyers in probation offices.

In order to participate in this study, participants had to be 18 years of age or older, conversant in English, and currently on probation in Hennepin County. The recruitment goal was not to achieve a random sample, but instead a purposive sample that maximized diversity across respondents (in terms of age, race/ethnicity, gender, criminal history, time on probation, health status, etc.). As described in more detail below, our final sample includes 162 adults on probation, with varied backgrounds and experiences. Participants were compensated for their time with a $40 honorarium and told that participating (or not participating) in the study would have no impact on their supervision. The interviewers also made clear that participants’ identities would be kept confidential and that nothing shared in the interview would be relayed back to their PO.^2^

Interviews were conducted by a team of undergraduate and graduate student research assistants (diverse across race/ethnicity and gender) who went through extensive training regarding confidentiality and consent, interview protocols, and data storage rules.^3^ Interviews took place in public cafés, libraries, and (when necessary) supportive housing facilities. The interview guide consisted of five modules: employment, housing, health, family, and criminal legal system experiences. The interview guide combined both closed-ended and open-ended survey questions in each module. The structured survey questions were modeled on the Boston Reentry Study (Western et al., 2017), validated physical and mental health screening tools, and substance use and healthcare access questions from the National Survey on Drug Use and Health (NSDUH). The interviews took approximately 1-2 hours to complete, with research assistants entering structured answers into an online interview software (Qualtrics) during the interview and capturing participants’ responses to open-ended questions through audio recorders. Research assistants later transcribed participants’ responses to open-ended qualitative questions.^4^ In the quotes below, we assign all participants a pseudonym.

Our analysis centers responses to the following question: “How do you think your current health compares to your health before you started probation?” with the response options: “Health is better now,” “Health is about the same,” and “Health was better before probation.” After the participant selected one of the options, the interviewer provided an open-ended qualitative prompt: “Please explain why you believe your health improved / worsened / stayed the same.” Out of a total of 166 participants who participated in the study, four participants did not answer this question. Therefore, we limit all analyses presented in this paper to the 162 participants with data on this question. Quantitative and qualitative analyses were completed simultaneously in a convergent mixed methods analysis (Hesse-Biber, 2010), with each informing the other iteratively as we analyzed the data. We examine both the characteristics of respondents in each category (better now, about the same, better before) quantitatively and the open-ended answers to the “Why” question. Across the results, we focused on trying to understand the personal, social, and structural circumstances associated with improved or worsening health on probation as well as how participants themselves understood the impact of supervision on their wellbeing.

For the quantitative analyses (conducted in STATA 14), we examined the correlations between the health change variable and a range of other measures from the study, including demographic variables (race/ethnicity, gender, and age), health and healthcare-related variables (self-reported health, history of substance use disorders and other health conditions, access to healthcare, and insurance status), material stability (employment, housing status, food insecurity, and receipt of public assistance), and experiences on probation (reporting frequency, perceptions of PO, and ratings of probation’s helpfulness and stressfulness). More detailed information about the quantitative variables included in the analysis can be found in Appendix. Across each metric, we examined the share of participants in each sub-category who reported improved, unchanged, or worsening health. We use chi-square tests to evaluate the hypothesis that each pair of categorical variables is independent (e.g., racial/ethnic categories and health outcome). We use a significant chi-square test result (at or below a *p*-value of less than 0.05) to indicate that there is a meaningful correlation between the two variables. For the key outcomes, we use stacked bar charts to visualize these correlations. Given the descriptive (rather than causal) nature of our study, and the fact that many of our variables are strongly correlated (e.g., past history of substance use and housing instability), we do not correct the *p*-value for multiple comparisons. Instead, we use significant results in an exploratory manner to highlight potential linkages between individual, social, and structural forces and individuals’ health.

For the qualitative analysis (conducted in NVivo 12), we followed Deterding and Waters’ (2021) flexible coding approach for in-depth interviews analyzed by a team, collectively developing a set of codes that included health-promoting behaviors, mental health, probation conditions, substance use and treatment, and social determinants of health (economic stability, education access and quality, health care access and quality, neighborhood and built environment, and social and community context.) Once the codebook was finalized, the second author coded the entire database in NVivo, with feedback from the first author on any points of confusion.^5^ Participants’ responses were coded to as many themes (nodes) as the response fit. For each quote, we selected the theme and its valence: “improved,” “worsened,” or “other” (for responses that were neutral, partially complete, or complex). We then collaboratively used the final codes to identify the most common themes and patterns. As we developed the analysis, to get deeper insights into participants’ stories, we picked individual quotes that exemplified each section’s themes and then went back to review that person’s entire interview transcript, building a more complete profile of their circumstances and experiences.

The findings section proceeds in four parts. First, we outline the demographic characteristics of our sample, variation in our key outcome variable (health improved/stayed the same/worsened since starting probation), and relationships between demographic variables and the health. Next, we consider the three themes or central pathways that emerged from our analysis: health, health-related behaviors, and healthcare services; material stability; and supervision demands and relationships with POs. Although all three of these themes overlapped in participants’ lives, for analytical clarity, we describe each separately.

## Results

Table 1 documents the characteristics of our sample. Across 162 participants, 36% identified as non-Hispanic Black alone, 38% as non-Hispanic white alone, 7% as American Indian or Native American, 3% as Latina/o/x or Hispanic, 6% as multiracial, and 11% as an other race/ethnicity. Three-quarters of our sample were men with a median age of 40.5 years.^6^ One-third of our sample had left formal education before completing high school or a G.E.D. Another 21% had completed high school or a G.E.D., while 26% had completed some college, and 22% were college graduates. The largest share of our sample (42%) was on probation for drug and alcohol-related crimes as their most serious offense, with another 30% under supervision for person-related crimes, and 18% for property offenses.^7^ Roughly a quarter (28%) of respondents had previously served time in an adult prison and they had served a variable amount of time on probation, from less than 1 year (44%), to 1 to 2 years (28%), 3 to 4 years (21%), and 5 years or more (6%).

**Table 1:** Characteristics of Study Sample and Self-Reported Health Change

	Percentage Distribution	Health Change Since Starting Probation		
		Health Better Before (%)	Health About the Same (%)	Health Better Now (%)
All respondents	100	20	25	55
Race/ethnicity				
Non-Hispanic White	38	23	18	60
Non-Hispanic Black	36	21	29	50
American Indian or Native	7	8	25	67
Hispanic or Latina/o/x	3	25	25	50
Multiracial	6	33	11	56
Other race/ethnicity	11	12	41	47
Gender				
Men	76	22	24	54
Women	24	15	26	59
Age				
Under 30 years	22	28	19	53
30-39 years	26	21	21	57
40-49 years	26	12	29	60
Over 50 years	26	21	29	50
Highest year of education				
Less than high school	32	18	25	57
12th grade or H.S. diploma	21	25	31	44
Some college	26	18	18	65
College graduate or higher	22	24	27	50
Most serious charge for conviction				
Drug or alcohol-related offenses	42	12	33	55
Offenses against persons	30	34	17	49
Property offenses	18	10	24	66
Other offenses	10	38	13	50
Years on probation				
Less than 1 year	44	25	32	43
1-2 years	28	10	25	65
3-4 years	21	30	13	57
5 years or more	6	22	11	67
N	162	33	40	89

Table 1 also displays the percent of our sample in each of the three primary health categories: 20% reported that their health was better before starting probation, 25% reported no changes overall in their health, and 55% said their health was better now. This health outcome was strongly correlated with participants’ aggregate mental health (*p* < .001) and aggregate physical health (*p* < .001) scores on the Short-Form Health Survey (SF-12), a validated measure of overall self-reported health (Ware et al., 1996). Thus, those reporting improved health were in better health than those reporting worsened health. However, there were no statistically significant differences between demographic characteristics (race/ethnicity, gender, age, and education level) and the share of respondents reporting worsening, unchanged, or unchanged health. For example, 53% of participants aged 18-30 years reported that their health was better now, compared to 50% among those aged 50 years or more. Similarly, 50% of non-Hispanic Black respondents saw their health improved, compared to 60% of non-Hispanic white respondents and 67% of Native participants.

### Health, health behaviors, and access to healthcare

**Fig. 1:**
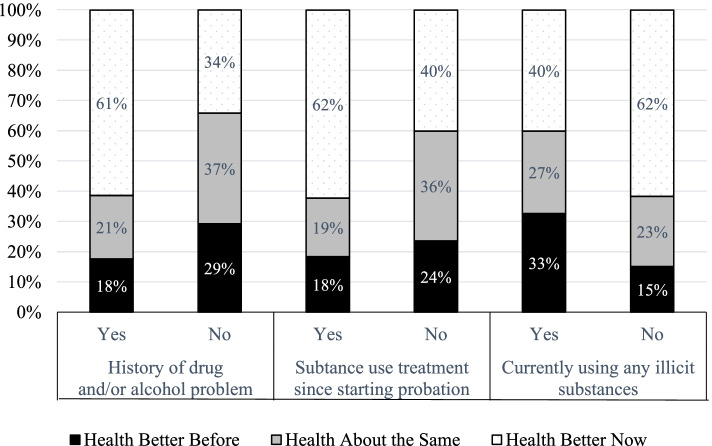
Substance Use, Treatment, and Health

One of the most consistent correlates of health across our sample, in both the quantitative and qualitative results, was sobriety, previous problems with drug and alcohol use, and substance use treatment. The majority of our sample reported a history of substance use problems (74%), which was associated with health changes during probation (*p* < .05). As displayed in Fig. 1, among people with a history of drug and/or alcohol problems, 61% reported improved health, compared to 34% without. For participants with prior substance use problems, most (75%) had participated in treatment since starting probation. In the majority of cases, this treatment had been mandated by the courts or probation. Hennepin County courts often require people convicted of criminal offenses to complete a “Rule 25” assessment, during which a health professional determines if an individual needs substance use treatment. Depending on the outcome of the assessment, and the person’s financial circumstances, many people on probation are able to receive state funding for treatment services (including in-patient programs). An overwhelming majority (85%) of those who participated in drug and/or alcohol treatment evaluated these services as “helpful.” Finally, attending treatment during the probation term was associated with better health (*p* < .05); Among participants who reported participating in substance use treatment since starting probation, 62% reported improved health, compared to 40% among those who had not participated.

Probation was also associated with a substantial reduction in substance use. In the 6 months leading to their arrest for their current term on probation, frequent illicit drug use (defined as twice a month or more) was reported by 51% of our sample for marijuana, 19% for cocaine/crack, 21% for methamphetamines, 11% for heroin, and 10% for prescription opioid misuse. In addition, 25% of participants reported drinking alcohol daily in the 6 months leading to their arrest. Rates of illicit drug use at the time of the interview were substantially lower, with more than half (64%) of the sample reporting zero use. By that point, only 15% reported frequent marijuana use; 12% frequent cocaine/crack, methamphetamine, heroin, or other opioid use; and only 2 participants reported daily alcohol use. Sobriety was associated with better health and, conversely, current substance use was associated with worse health. For example, participants who reported drinking any alcohol at the time of the interview were more than twice as likely to report worsening health, compared to those not drinking (35% vs. 15%, *p* < .01). Similarly, as displayed in Fig. 1, participants currently using illicit drugs were twice as likely to report worsening health (*p* < .05).

In the quantitative results, other health-related characteristics mattered less than substance use and treatment for health outcomes during probation, but similarly documented the stark needs facing adults on supervision. At some point in their lives, most participants (71%) had been diagnosed with a mental health condition, a history associated with health outcomes (*p* < .01). Among people with a history of mental health diagnoses, 59% reported improved health, compared to 44% of those without such a diagnosis. However, people with a mental health diagnosis were also somewhat more likely to report worsening health (22% vs. 13%), suggesting a bi-modal distribution. Over half of participants (66%) had seen a mental health professional since starting probation, though having had these visits was not significantly associated with health outcomes. Most of our participants (68%) also had a history of chronic physical health conditions, including diabetes, high blood pressure, or asthma, and had seen a doctor since starting probation (85%). Yet having received physical health diagnoses in the past was not reliably correlated with improved or worsened health, nor was receiving medical care (not including drug or alcohol treatment) since starting probation. Finally, somewhat surprisingly, whether or not participants consistently had health insurance coverage over the past year was not significantly correlated with improved or worsened health.

When participants who reported improved health were asked “Why?” the predominant qualitative theme (reported in roughly half of our interviews) was the reduction in substance use we saw in the quantitative results. Sobriety was often, though not always, mentioned in conjunction with attending substance use treatment programs. In addition, most participants were subjected to random drug tests (organized around a “color wheel,” in which the color assigned by one’s probation officer represents how frequently the person should expect to have their color selected and a drug test required). Forced by the court and/or probation to end (or reduce) their substance use, many participants found themselves embracing other positive health behaviors. Leonard, a 52-year-old white man who started substance use treatment after beginning probation, told us how his new-found sobriety allowed him to meet basic needs:

“When I was using, I would be living off a Super America or Holiday hotdogs … I was just eating a lousy diet, lousy sleep, lousy living conditions, just not taking care of my daily hygiene needs and stuff … Now I'm eating way healthier. Changed my whole diet, you know, hygiene's great, stuff like that. Just, you know, people take for granted that when you're in the horrors of addiction.”

For Leonard, sobriety was tied to a bundle of changes in his ability to care for his body and mind, including a safe place to sleep.

Like Leonard, many other participants credited substance use treatment and their new sobriety with improved health. Jessica, a 26-year-old white woman, had been struggling with substance use for years, with several convictions to prove it. She described: “I was an IV heroin and meth user, so my life was centered around my drugs … my health was not important and definitely wasn’t a priority like it is now … I was almost destroying myself.” Jessica was mandated to substance use treatment while on this term of probation, and felt that it was helpful: “I did and I do have a substance abuse issue, so treatment has helped me kind of get my life back together and have some, you know, structure.” Jessica reported that she is now scheduling health appointments and making her wellbeing a priority, which she attributed to her sobriety. Since the time of her arrest, Jessica had seen a healthcare professional a few times a week and accessed a variety of different health services. As a result, Jessica described her health as having improved since starting probation.

For some participants, this new access to care included addressing long-standing health concerns, including depression and anxiety. Donna, a 43-year-old Black woman, attributed much of her recent strides towards good health to substance use treatment and counseling. While Donna had experienced many previous encounters with the criminal legal system, this was the first time she had been offered treatment. Donna was mandated to dual-diagnosis treatment to address her substance use and mental health conditions, which entailed attending substance use treatment programming, a trauma group, psychiatric care, and therapy. This was a pivotal experience for Donna, allowing her to address a lifetime of trauma and learn coping mechanisms: “I’m learning how to deal with it and talking about it versus not talking about it and doing drugs. It’s hard but it’s something I can do.” After years of criminal legal system contact, accessing treatment allowed Donna to address the causes of her substance use and gave her the support she needed to “want to change my life.” Additionally, after getting on Medicaid, Donna was able to supplement the health gains she made through treatment by accessing other healthcare services, including primary care and dental services.

These gains, however, were hard-won and vulnerable to disruption. Leonard, for instance, still struggled with a desire for substances, describing staying drug-free as the most difficult part of supervision and calling it a “vicious battle.” Leonard compared “staying clean” with the movie *300* (which portrays a fictionalized account of the Battle of Thermopylae in the Persian Wars), declaring: “It’s like being on the front line of a hand-to-hand combat with swords and you know I gotta constantly be on guard.” Other respondents described how they had gotten sober in the past, only to relapse on substances. Marcus, a 27-year-old Black man, described how he had continued to have relapses with his substance use, which he characterized as an on-and-off cycle: “I try to be sober, it’s not like I don’t make an effort to be. I just continue to have relapses.” Like many others, Marcus was mandated to attend substance use treatment, but he was kicked out of this program after getting sober (ostensibly for “threatening” a staff member, though Marcus disputed the claim). As a result, Marcus lost his housing (which treatment was paying for), destabilizing his progress and precipitating another stint in jail. As Marcus recounted:

“I lost my housing, and was back out on the street, so I just said fuck it and I got high for like five days… I got so high, and I came down, and I just couldn't get up to his office. It was just a bad deal. And I didn't know he wanted me to go, I had no phone. I knew I was in trouble, so I just didn't go check in at all for a couple weeks. And then I got picked up and that was it. So I was in jail for like 20 days.”

This was one of many attempts at sobriety Marcus had made on probation and during previous stints in prison, each of which was eventually derailed.

For people like Marcus, testing and treatment mandates did not always produce sobriety—and, at times, could prompt worsening health. Among the 33 participants who reported worsening health since starting probation, answers to the open-ended “Why?” question again often invoked substance use, this time to say that continued use and the strains of supervision were negatively impacting their health. For example, Marcus described in detail how routine drug tests and treatment “have not been effective” in helping him manage his addiction and the costs of conviction and supervision on his wellbeing: “I used to exercise all the time. Like I said, I had my own shit and my own place, you know what I mean? I had my car, I had my shit, I had money, I had fucking whatever. I used to exercise all the time… all the time.” Now, Marcus continued, “I just haven’t had shit… it’s hard to stay in shape and I’m still doing drugs sometimes.” Marcus said he has not been able to stay drug and alcohol-free while on probation; he considered both supervision and his drug addiction to be significant barriers to his wellbeing.

For a small number of participants, coerced sobriety had worsened their health by removing a coping mechanism, including pain management and emotional regulation. Martin, a 51-year-old white man with an extensive list of health conditions, including a chronic pain condition, told us that although he had gotten sober, he felt his health had gotten worse as a result of not being able to self-medicate:

“When I was actively using drugs, I wasn't feeling the pain … Soon as I sobered up and went into recovery again, is when my symptoms start showing up more … when I was high, I could move around, I wasn't feeling the pain … So … it feels worse.”

Probation had coerced Martin to get sober, but had not helped him address the chronic health condition underlying the drug use. Other participants also described how their fear of a positive drug test led to negative compensatory behavior, including drinking alcohol more frequently and smoking cigarettes. This was particularly true for people who previously used marijuana to self-medicate.^8^ Esperanza, a 42-year-old Latina, used to smoke marijuana on a regular basis. Now that she was required to submit monthly drug tests (or “UAs,” which stood for urinary analysis) while on probation, she began drinking alcohol and smoking cigarettes more heavily instead. She made this switch because she wanted to make sure that her UA tests were negative. Esperanza said that quitting marijuana was the hardest part of probation for her and now, she’s “drinking all the time,” prompting worries about her health. Ethan, a 28-year-old Black man, similarly described substituting marijuana for K2, a synthetic version of marijuana, because it did not show up on his UA. The K2, however, disrupted his digestive system, ending in a diagnosis of cyclic vomiting syndrome.

In sum, participants who accessed healthcare, especially substance use treatment, and were able to change their health behaviors, experienced improved health on supervision. These gains were often attributed to supervision indirectly rather than directly—by mandating sobriety and referrals to services, probation provided coercive motivation to address this health issue. But the meaning of substance use (and abstinence) varied across participants, from a “risky” health behavior and potential criminal offense to a coping mechanism and/or treatment for a medical condition. As a result, for some participants (roughly 1 in 5), their health had worsened while on probation. In addition, as we describe below, participants without a minimum level of stability could not expect to complete treatment and probation requirements, putting them at risk of compounding punishment through revocation.

### Material stability: housing and food insecurity

As seen in the cases described above, sobriety and improved health was often connected to increased material security, including safe housing and access to healthy food, while precarity was associated with substance use and worsening health. Our sample faced substantial material insecurity, often despite the receipt of state aid. The majority of participants were not employed (61%), with just under half (46%) receiving public benefits (including food stamps, housing assistance, and/or income assistance). Nearly half (43%) reported that it was “slightly” to “very” difficult to provide themselves with food, a broad measure of food insecurity.^9^ In addition, only 24% of respondents lived in their own home or apartment at the time of the interview, with the largest share living in supportive housing (35%) or with friends, partners, and relatives (35%).

**Fig. 2:**
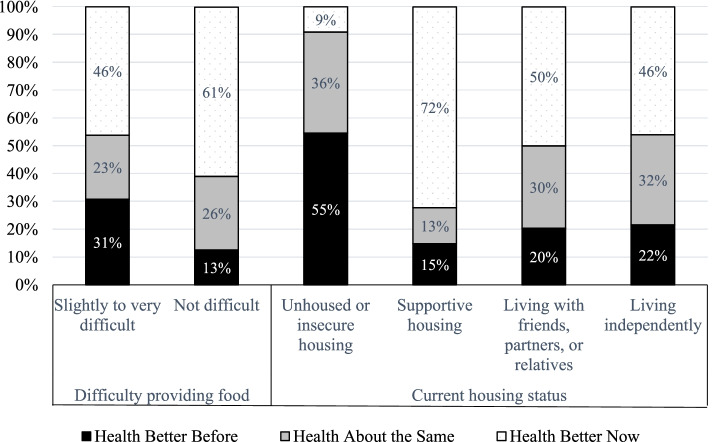
Food and Housing Security and Health

The social determinants of health framework posits that individuals’ health and wellbeing are tied to structural inequalities, including economic opportunities and the built environment. Among our participants, employment status and receipt of public assistance programs, however, were not significantly associated with differences in self-reported health. In contrast, food insecurity—perhaps a clearer measure of ongoing economic need—was strongly associated with participants’ perceptions. As displayed in Fig. 2, respondents facing food insecurity experienced more than twice the rate of worsening health (*p* < .05). In addition, there was a strong coupling between housing status and health (*p* < .01). Only 9% of those with insecure housing reported improved health, a share drastically lower than the sample mean. In contrast, the highest percent of respondents reporting improved health (72%) was among participants living in supportive housing, followed by those living with friends, relatives, or partners (50%) and those living independently (46%).

Looking in more detail at respondents in supportive housing, most participants were living in in-patient drug treatment programs, sober living houses, and half-way homes. Most participants stated that they had received “support finding or obtaining” this housing. Some participants had been mandated to these facilities (e.g., court requirements to complete in-patient treatment) or found housing through assistance from their PO, while in other cases, participants accessed housing through a social worker or referrals from treatment programs or jail and with support from government programs. As a result, the transition to probation was associated with a substantial reduction in experiencing homelessness: whereas a fifth of our sample was living on the streets, in hotels/motels, or “moving around a lot” at the time of arrest, only 7% were living insecurely at the time of the interview. More broadly, a quarter of our participants reported that their PO had helped connect them to assistance programs like housing vouchers, food stamps, and/or disability aid.

In the qualitative responses to “Why?” their health had improved, stayed the same, or worsened, participants often connected their material stability (especially housing) with their current health. As described above, these discussions were often tied to sobriety or substance use. As Owen, a 29-year-old biracial man who was living at a sober living house, noted: “When you’re on the streets you worry about life; where your next meal going to come from, where you’re going to sleep at, especially if it rains or snows, you know the weather up here. Now I ain’t got to worry about that, I got a place to lay my head, eat, shower, everything.” Similarly, Jasmine, a 21-year-old Native and African American woman, felt that obtaining housing was vital in helping meet her basic needs (including food insecurity) and improve her health:

Jasmine: “I don't want to say I was malnourished, but I definitely wasn't eating the way I was supposed to because I was homeless, like I was losing a lot of weight.”

Interviewer: “So you see yourself eating better now that you have housing?”

Jasmine: “Yeah.”

Although Jasmine still faced many problems, including extremely low income and multiple chronic health conditions, housing had a large impact on improving her health overall.

Jasmine’s story also illustrates how mandates to treatment and coerced sobriety could backfire when people lack material stability. Jasmine was required to call in daily to learn whether she would be expected to come in for a drug test that day. Before finding the housing described above, Jasmine was living on the streets and did not have a phone so was not able to call in. Because of this, she missed UAs, which were counted as positive, which resulted in a probation violation. As a consequence, Jasmine was mandated to undergo substance use treatment. Despite feeling like she didn’t need treatment (the only substance she reported using was marijuana and Jasmine did not identify that use as a problem), Jasmine felt that this out-patient treatment program was helpful because they had food shelves and free clothing. However, Jasmine stopped attending this program in winter, when waiting for public transportation in extreme winter temperatures without an appropriate jacket became untenable. As a result, she was kicked out of the program and faced another violation. At the time of our interview, Jasmine had found housing through a voucher program and was awaiting a chemical dependency assessment. Without addressing the material instability that Jasmine faced, mandated treatment requirements were not sustainable.

On the other end, for participants who had some measure of material security before probation, their arrest, conviction, and supervision could set off a cascade of consequences that worsened health. While most participants had very little income at the time of arrest, roughly 1 in 10 held a job with benefits. For these participants, their arrest, conviction, and supervision could trigger and/or exacerbate a downward spiral. Jennifer, a 36-year-old white woman, was a teacher before her arrest and lived in her own place. She had recently lost her father and was in an abusive relationship with a man with whom she had two children. Jennifer told us this boyfriend had PTSD from time in the military and had tried to kill her during one fight. By the time of the interview, Jennifer had been arrested and convicted of disorderly conduct (for what her ex had claimed was assault during an altercation). The arrest, legal processing, and transition onto probation led Jennifer to lose her job, eventually moving in with her mother. She noted that she had multiple health conditions (including depression, anxiety, and high cholesterol) that had worsened since starting probation, and when asked why, expressed how her struggles to find both employment and housing with a criminal record impacted her:

“I can't get a freaking job because of my probation and my criminal background … I don't have my own living situation anymore after years of my own living situation. I'm more broke … and then there's just like too much stress that's affecting everything … It's just all added up, and I don't have one area of my life where I really have freedom or like happiness associated with it because it's just like so much that I can't do.”

For Jennifer, who had some stability before the arrest, her criminal conviction and probation sentence had prompted significant downward mobility, which in turn worsened her health.

In addition, in-patient treatment programs, sober houses, and living with friends and relatives, were often temporary solutions to long-term problems. For example, Owen, the 29-year-old biracial man who above described the benefits of living at a sober living house, described the facility as “unsanitary” and stated that the neighborhood was an unsafe place to live. Owen also expressed concerns about having a roommate who drank heavily and was looking to get transferred as a result of these difficulties. At some point, people had to leave these contexts and find independent housing, often with little support and facing steep barriers, including housing discrimination against people with records and limited affordable housing in the metro area (Quirouette et al., 2016). Many participants spoke at length about the stark realities of looking for housing as people with criminal records and the constant denials they heard from landlords (see also Herbert et al., 2015). Jennifer, the 36-year-old white woman mentioned above who discussed her downward mobility since starting probation, described how she had stayed in about 20 different places since her arrest trying to find a home for her family: “I’ve tried to figure out where I can rent because they also do criminal background checks, so I’m like, shoot, I cannot figure this out.’” Thus, the material gains and health improvements provided by in-patient treatment programs, half-way houses, and sober living facilities were often only a temporary intervention rather than a structural solution.

### Supervision demands and relationships with POs

As described above, when asked why their health had worsened, stayed the same, or improved, participants rarely spoke directly about their POs. Instead, they underscored the broader circumstances of their lives like sobriety or substance use, access to healthcare or treatment programs, food insecurity, and housing. At times, the link between supervision and these circumstances was clear, for example, when POs mandated or referred people to drug and alcohol treatment programs, housing assistance, and other services. This interpersonal dynamic and service referral, however, was only part of supervision. In addition to the criteria set out by the courts at sentencing (including, for many, mandated substance use assessments), the department had its own set of policies, including things like standard probation conditions and rules about how to identify which cases merited more intense supervision. In this final section, we turn to how participants described their relationships with POs and the conditions of supervision more broadly to unpack the relationships between these demands and self-reported health.

**Fig. 3:**
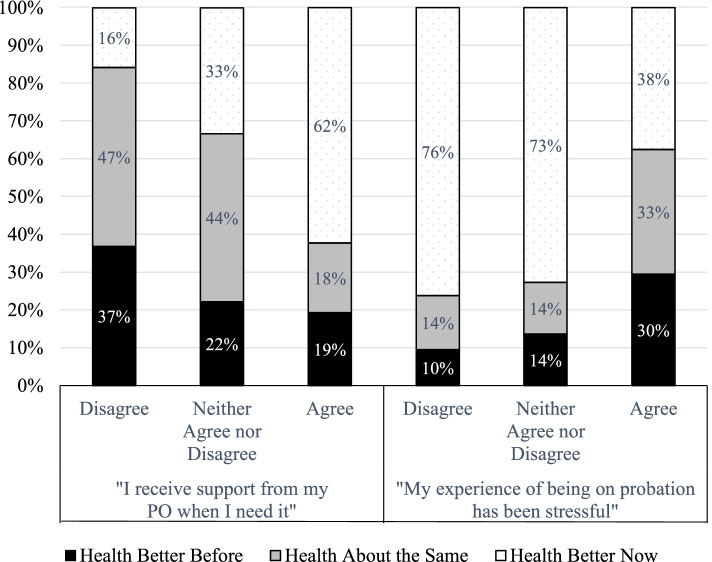
Supervision Experiences and Health

We turn first to a battery of questions about participants’ perceptions of their POs. The vast majority (90%) of participants agreed that “My PO treats me fairly,” while 75% agreed with the statement “I receive support from my PO when I need it.” Respondents with more positive evaluations on both of these relationship indicators were more likely to say their health had improved since starting probation (*p* < .01). As displayed in Fig. 3, among participants who selected “agree” or “strongly agree” to the statement “I receive support from my PO when I need it,” 62% experienced improved health, compared to 16% among those who selected “disagree” or “strongly disagree.” Similarly, the majority of respondents (77%) who reported that probation had been “somewhat” to “very” helpful were significantly more likely to say their health improved (*p* < .01), with 70% of participants who reported that probation had been “very helpful” reporting better health, compared to 37% of those who reported that probation had been “not at all” helpful. Notably, just over half of participants (55%) reported that their PO had asked about their health, an interaction that was associated with improved self-reported health (*p* < .001). A smaller share (16%) reported that their PO had helped connect them to health services, which similarly was associated with a higher likelihood of improved health (*p* < .05).

Qualitatively, relationships with POs looked different across the interviews. When asked to describe the relationship with their POs, participants expressed a wide range of sentiments. Some participants felt as though their relationships with their POs were “nonexistent,” while a handful spoke about deeply negative relationships with their POs. These POs were perceived to be untrustworthy, intimidating, overly subjective in imposing sanctions, and judgmental. The majority of participants, however, described their POs positively, citing clear and transparent communication as a key factor in fostering positive relationships. When POs were understanding of participants’ circumstances and fair in imposing sanctions, participants felt as though they could be more honest with them and build a trusting relationship. Notably, support from POs was most commonly experienced when POs served in a social worker role, connecting individuals to resources and services. This kind of “therapeutic alliance,” or positive relationship between the client and PO, is a key component of how probation might provide interventions tailored to individuals’ needs (Bourgon & Guiterrez, 2013). Many participants described these referrals as a form of “care,” with those programs and resources helping participants to reach greater material security as described above (see also Welsh, 2017). The most common referral was to substance use treatment programs, but participants also described how their POs had referred and/or mandated them to other health services, assistance programs (e.g., food stamps, housing vouchers, or disability), educational programs, and other resources.

In addition, the PO-client relationship was structured by the broader conditions of supervision, constraints some participants experienced as coercive motivation for improving their health. For example, participants who rated probation as “very helpful” often described that the court and probation process pushed them to “walk the straight and narrow” and served as a “wake-up call.” Darius, a 25-year-old Black man, explained how probation was helpful to him:

“I feel like, you know, me being on probation and me not being out like I was before I got on probation, you know what I'm saying, just being reckless. Not having no type of care in this world, nothing. Now that I'm on probation it actually gave my health a little bit of breeze, took a little stress off my shoulder, made me feel like I'm more clear-headed.”

Similarly, Donna, who accessed substance use treatment and counseling services while on probation, felt that her experience on probation had been “very helpful”:

“It keeps me criminal free … It gives me a routine, productive routine. And helps me to better myself … you know, keeping me out of the criminal system. And it helps me access resources that I need.”

For participants like Darius and Donna, and many of the others described above, the constraints of probation enabled them to move towards more positive health behaviors and increased material security.

Yet this coercive motivation had a double-edge. While participants like Darius found supervision focused their attention, he also reported that supervision was “very stressful.” While probation might have been a “wake-up call,” Darius also detailed the difficulties and collateral damage that came with a criminal record. At the time of the interview, Darius relied on government programs to support himself and had an impossible time searching for jobs with his criminal record. He reported that this had made it “very difficult” to support himself financially while on probation. The onerous conditions of probation were also stressful for him to navigate, especially when he had to catch multiple buses to make his appointments on time. While Darius was confident that he was “not doing nothing that’s going to lead to actually getting a probation violation,” he still expressed that he was scared of going back to court and being sent to prison.

**Fig. 4:**
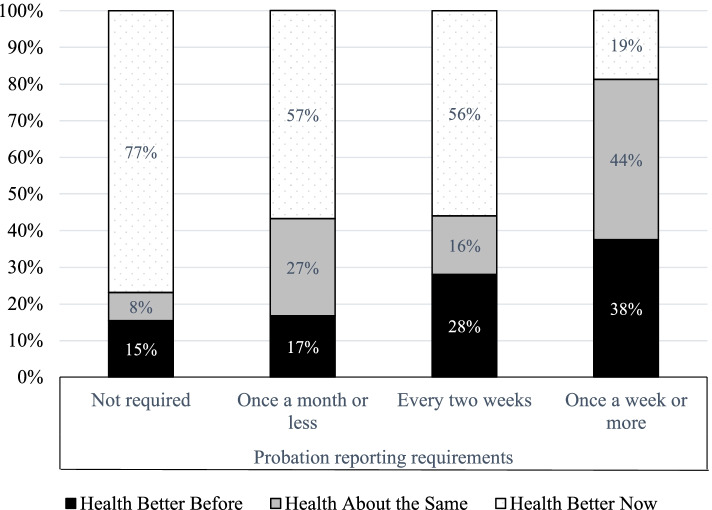
Reporting Demands and Health

For some participants, these costs of supervision outweighed the benefits. More than half (57%) of participants agreed or strongly agreed with the statement “My experience of being on probation had been stressful.” These respondents were less likely to see health improve and more likely to see health declines (*p* < .001). As displayed in Fig. 3, among participants who agreed with this statement, 30% reported better health before probation, compared to only 10% among those who disagreed that probation was stressful. Experiencing supervision as stressful was also correlated with reporting frequency (*p* < .05). Across our sample, 8% were not required to report regularly, 65% checked in once a month or less, 16% twice per month, and 10% once a week. As displayed in Fig. 4, 38% of those who reported to their PO once a week or more reported worsening health, compared to roughly 15% among participants who were not required to regularly report to a PO (“paper only” supervision) or who reported once a month or less. Supervision stress was also correlated with participants’ perceptions of the likelihood of violation (*p* < .0001); Those who rated it as “difficult” or “very difficult” to avoid a probation violation since starting probation were significantly more likely to agree that probation was stressful, while those who found it “not at all difficult” or “slightly difficult” to avoid violations were much more likely to disagree that probation had been stressful. In addition, these perceptions of the difficulty of avoiding violations and the stressfulness of probation were correlated with continued illicit substance use (*p* < .01).

Respondents weren’t wrong about this potential threat of incarceration. Indeed, in a study of POs in Hennepin County conducted during the same time period, the sampled POs reported that they recognized the high rates of substance use disorders, other health challenges, and overall precarity of their clients. Yet two-fifths said in response to a vignette study that if a person on probation was testing positive for opioid use, they would likely tell the client “he’s going to end up back in jail.” A quarter of POs said they would bring the person in for a court appearance to show the threat of revocation for drug use. Officers were similarly severe in response to a vignette case where the person had stopped taking their prescribed mental health medications and skipped appointments with a mental health professional (Mitchell et al., 2021). In addition to highlighting the prevalence of revocation threats, these findings also highlight the variability in PO responses that adults had to navigate in the system.

In the open-ended replies to why their health had changed, roughly half of our respondents who reported worsened health mentioned the burdens of supervision on their mental health (see also Semenza & Link, 2019). Mental health was almost always discussed in conjunction with probation conditions, including the risk of revocation and the demands of enforced sobriety and strict reporting requirements. Ethan, the 28-year-old Black man who above described how substituting other substances for marijuana impacted his health, expressed the toll that probation had taken on his mental health: “I was in a more positive state of mind before probation… it’s always a worry.” Ethan has multiple mental health conditions, including depression and anxiety, which were diagnosed after starting probation. Ethan was also one of our participants who was able to find employment, but he still relied on family support to survive, including living with his mother. In the interview, he described how supervision exacerbated his mental health challenges: “With anxiety I’m always worried about my freedom, ‘cause in probation it’s, um, from past experience I could go to jail at any minute just because of probation and not even know why, it’s just all up to him, my probation officer, of my freedom.” Having been returned to custody before, Ethan felt it was “very difficult” to avoid a probation violation, noting that any kind of deviation from his PO’s conditions could mean a revocation. At the same time, Ethan reported, his PO had done little to connect Ethan to the kinds of services that would make a difference. In contrast to some participants, Ethan had completed substance use treatment during probation, but found it “pointless” and an active impediment to “daily routines.” For adults like Ethan, probation was a trap—giving him the illusion of freedom, while in reality imposing significant and painful constraints.

Jessica, the 26-year-old white woman who described how getting sober had allowed her to focus on her health, also reported that probation was quite stressful. Despite having an amicable relationship with her PO and being mandated to substance use treatment, which she felt was “life-changing”, Jessica still described probation (which in her case was tied to participation in drug court) as being “held to a standard I can’t meet.” Similar to Ethan, Jessica reported that her mental health conditions had been exacerbated by probation: “There’s a lot of obligations to fulfill and things to do for drug court …I have a lot of anxiety just, you know, wanting to do good or do better and not let my probation officer down, cause they’re pretty strict.” She also mentioned that her anxiety has worsened due to a constant fear of revocation, which was the most difficult part of probation for her. Even though probation helped connect her with a healthcare provider, supportive housing, and substance use treatment, those benefits were tempered by stringent probation conditions, lofty expectations, and the constant threat of revocation, which drastically impacted Jessica’s quality of life and mental wellbeing. As Jessica described, “if you like miss a UA because you’re sick or something like that it’s like BOOM they can put you in custody.” Despite maintaining her sobriety, Jessica had recently faced a punishment of extra community service days after testing positive for marijuana three times, a result of her use of CBD oil she thought was legal for her to use, an error revealed to her only when her PO brought her in front of a judge for a violation hearing.

Jessica’s ambivalence about supervision was common across our sample, with a majority reporting that supervision was both “helpful” and “stressful.” Where people benefited from supervision, it was as often less about the actual demands and “care” of supervision than it was about the services and resources their PO helped them to access. These resources, whether it be substance use treatment programs or housing assistance, allowed participants to begin to address the many barriers they faced to thriving in the community. In contrast, the harms of supervision for health came predominantly from supervision itself—and, more specifically, the threat of revocation. As Ethan aptly summarized: “I’m always worried about my freedom.” In addition, these costs were often born by people in deeply precarious socio-economic contexts, for whom probation was just one of many negative encounters with state authorities.

## Discussion

In this article, we used an interview study with 162 adults on probation in Hennepin County in 2019 to ask: (1) How do adults on probation report their health has changed since starting probation? (2) What are the social and structural factors or mechanisms associated with improved or worsened health on probation? We found that roughly half of our participants described their health as improving since starting probation, with the other half split between no change and worsening health. Exploring the relationship between this health status change and individuals’ life circumstances quantitatively, we find that there were few statistically significant correlations between demographic characteristics (age, race/ethnicity, education, and gender) and self-reported health improvements. However, people with a history of substance use issues were more likely to report improved health, in large part mediated by access to treatment while on supervision. We also find strong associations between improved health and food security and supportive housing. Finally, participants who had more trusting and supportive relationships with their probation officers, and experienced supervision as less stressful, were more likely to report improved health. Conversely, those with poor relationships and greater stress from supervision were more likely to see their health deteriorate.

Turning to the qualitative results from open-ended prompts, we find that when asked “why” their health had improved or deteriorated, participants often spoke of substance use treatment and sobriety, on the one hand, and stress from the fear of revocation, on the other. Sobriety or drug/alcohol use was rarely experienced in isolation–instead, struggles around substances were tied to other forms of precarity (including housing and food insecurity) and health challenges (including mental health conditions and chronic pain). This dominance of substance use talk among our sample is perhaps not surprising in a context where the majority of people on probation in the county had struggled with drug and alcohol use disorders (Olson et al., 2021). Yet participants also spoke powerfully about the stark limits and binds of supervision: forced to report to the office, submit to random drug tests, and more, some of our most vulnerable participants described the impossibility of avoiding violations. For those participants, probation was a temporary and illusory period of freedom, interspersed between stints of incarceration. As experienced by people on supervision, these burdens of supervision could negatively impact physical, mental, and behavioral health.

Our study has notable limitations. First, we can only generalize to participants in our study, which were a particular subset of all adults on probation in Hennepin County. Perhaps most importantly, eligible participants had to be actively on supervision, which means that people who had been revoked were ineligible for the study (though many of our participants had experienced a prior violation). In addition, while we asked participants retrospective questions, the study was not longitudinal, preventing long-term follow-up and an opportunity to see how health changed prospectively over the period of probation. The most significant health impacts of probation may be long-term and/or concentrated amongst the people who fail on probation and wind up behind bars instead of in the community, dynamics we hope other scholars investigate. Nor was our study designed to disentangle the likely bi-directional relationships between health and supervision (i.e., the impact of better or worse health on perceptions of supervision) or to provide causal impacts of probation on wellbeing. By centering the lived experiences of people on supervision, however, we have shown how any causal relationships between probation and health are likely to be difficult to estimate given the multiple and contradictory pathways.

This paper also complements the growing body of literature on the experience of supervision by focusing attention on the potential health-related impacts of supervision. Consistent with Phelps and Ruhland (2022), we find that probation provides both pains and gains, connecting people to services and providing a form of coercive motivation, while also burdening precarious adults with significant demands and new legal risks. As work by legal scholars demonstrates (e.g., Klingele, 2013; Doherty, 2016), our participants experienced this legal risk as a form of limbo, with one foot in the community and another behind bars (see also McNeill, 2019). In addition, the “gains” offered by probation were often coercive and punitive—doling out aid alongside punishment (or “pains”). Many of the programs that participants found relief through (including in-patient treatment programs and transitional housing assistance) were explicitly temporary, providing a meager form of state assistance conditional on hard-to-meet stipulations like remaining free of substances and any contact with police. When a person on probation achieves stability in a sober living house, for example, they are still bound by the rules and requirements of the housing facility (Rosenberg et al., 2021) and expected to later find independent, affordable, and safe housing, despite the many structural barriers that typically prompt contact with the criminal legal system in the first place. Failing to meet these requirements, or simply reaching the end of their period of eligibility, many participants recognized they would be facing an unforgiving housing and labor market on their own—with all the likely health consequences that follow that insecurity.

## Conclusion

Evaluating the high mortality risk faced by adults on probation, Wildeman et al. (2019) conclude that public health interventions must be developed for this “most overlooked, criminal justice-involved population” (661). To develop such interventions, however, we must understand the complex (and contradictory) potential relationships between supervision and poor health. Our research suggests that addressing the health needs of vulnerable adults through coercive interventions is unlikely to address the broader social determinants of health and may in fact deepen precarity.

Indeed, to the extent that participants found probation “helpful,” it was often the help *outside* of the criminal legal system that mattered most. And yet, people understood that they had to put up with the coercion of the state in order to receive (temporary, often punishing) assistance with basic needs like food, housing, and healthcare. Further, these services were often fragmented (Smith et al., 2019) and difficult to reconcile with the demands of supervision. This help then often trapped people in a cycle of poverty and insecurity, falling through a series of revolving doors in the penal and welfare systems (Halushka, 2020; Paik, 2021). Instead of deploying supervision, coercive threats, and revocation to respond to people facing material insecurity, chronic physical and mental health conditions, and substance use disorders, we join a growing chorus in arguing that many of these needs would be better met through reductions in the probation population (Lopoo et al., 2022) and substantial community investments (Hawks et al., 2021), including financial and housing assistance (Holtfreter et al., 2004; Hamilton et al., 2015) and affordable healthcare (Simes & Jahn, 2022). These programs also need to be designed to explicitly consider racial inequities, in addition to class disparities, if they are to ameliorate rather than exacerbate structural racism (Hardeman et al., 2021).

In the context of the COVID-19 pandemic and the policy changes that emerged to respond to the risk of the virus, some of these changes perhaps seem easier to imagine. The pandemic substantially altered the experience of community supervision, prompting many departments to radically reduce in-person visits, drug testing, and revocations in an attempt to slow viral spread (Kaeble, 2021; Powell et al., 2022). In this context, Hennepin County, our research site, shifted to using drug tests to support sobriety rather than as compliance monitoring, with treatment as the first response to positive drug tests rather than violations (Gokey, 2021). They also moved to redeploy some department resources from supervision to supportive services like housing. As counties and states reimagine a new “normal” in the wake of the COVID-19 pandemic, supporting these kinds of investments can help individuals, families, and communities reach their full potential.

## Data Availability

The datasets analyzed in the current study are not publicly available due to confidential participant information included in the interview transcripts.

## Appendix

**Appendix:** Data Description

Variable	Description
Health change since starting probation	Participants were asked “How do you think your current health compares to your health before you started probation?” Response options included: “Health is better now,” “Health is about the same,” and “Health was better before probation.”
Race/ethnicity	A categorical variable describing a participant’s race/ethnicity. This variable was created by combining one survey question about race and one survey question about ethnicity.
Gender	A categorical variable describing a participant’s gender identity.
Age	A categorical variable describing a person’s age.
Highest year of completed education	A categorical variable describing the highest grade or year of school that participants completed.
Most serious charge for conviction	A categorical variable describing the most serious criminal convictions that led to a participant’s probation sentence.
Previously served time in adult prison	A binary variable indicating whether a participant had previously spent time in an adult prison.
Years on probation	A categorical variable describing the length of time (in years) that participants spent on probation at the time of the interview.
Self-Rated Health	A categorical variable describing a participant’s self-rated health, with choices ranging from “Poor” to “Excellent”.
SF-12 Aggregate Physical Health	A summary measure of participants’ physical health, which is derived from responses to the Short-Form Health Survey (SF-12). SF-12 scores fall within the range of 0 to 100, with higher scores indicating better physical health.
SF-12 Aggregate Mental Health	A summary measure of participants’ mental health, which is derived from responses to the Short-Form Health Survey (SF-12). SF-12 scores fall within the range of 0 to 100, with higher scores indicating better mental health.
History of mental health condition	A binary variable indicating whether a participant reported a previously diagnosed mental health condition.
Visited a mental health provider since starting probation	A binary variable indicating whether a participant had accessed mental health services since the time of the arrest that led to their current term on probation.
History of substance use problems	A binary variable indicating whether a participant reported a history of substance use problems.
Substance use treatment since starting probation	A binary variable indicating whether a participant had attended substance use treatment since starting their current term on probation.
Currently using any illicit substances	A binary variable indicating whether a participant reported current use of any illicit substances (including marijuana, heroin, cocaine or crack, methamphetamine, prescription pain medication, prescription sedatives, and/or injecting drugs with a needle).
Currently drinking alcohol	A binary variable indicating whether a participant reported current use of alcohol.
Health insurance coverage over past year	A binary variable indicating whether there was a time in the past 12 months when a participant did not have any health insurance coverage.
Employment	A categorical variable describing a participant’s employment status at the time of the interview.
Receiving public assistance	A binary variable indicating whether a participant was currently receiving public assistance (including food stamps, WIC, welfare, general assistance, emergency general assistance, SSI, Minnesota Supplemental Aid, SSDI, unemployment insurance, housing vouchers, and other public housing assistance) at the time of the interview.
Food insecurity	A binary variable indicating whether a participant was currently experiencing food insecurity. If participants reported that it was “Slightly Difficult,” “Difficult,” or “Very Difficult” to provide themselves with food while on probation, they were considered to be experiencing food insecurity.
Current housing status	A categorical variable describing a participant’s current housing situation.
Probation reporting requirements	A categorical variable describing how often a participant was required to report to (or contact) their probation officer at the time of the interview.

## Footnotes

1 As of 2020, only 4% of adults on probation were reported to be under “inactive” supervision, which means that the person has no active reporting requirements (Kaeble, 2021). This is likely an under-count, however (Huebner & Shannon, 2022; Phelps, 2017).

2 As explained in our consent form, the one exception to confidentiality would be for mandatory reporting for ongoing abuse of children or elderly adults and/or specific plans to harm oneself or others. There were no cases where confidentiality needed to be breached by the research team.

3 The research protocol was approved under the University of Minnesota’s Institutional Review Board.

4 For roughly ten participants, transcripts were unavailable due to corrupted audio files, inaudible voices, or participants not consenting to be recorded; in these instances, we coded the interviewer’s notes.

5 Due to the thematic nature of the coding (rather than evaluative), we did not conduct inter-rater reliability tests (McDonald et al., 2019). In addition, inter-rater reliability can be difficult to conduct on semi-structured interviews where the length of text in different passages varies. Our analysis and interpretation do not rely on the counts of distinct thematic codes. Instead, the coding allowed us to identify broad trends and then select individual participants to highlight in each section.

6 While the gender and racial composition of our sample is roughly comparable to the population of adults on probation in the county, the percentage of middle-aged and older respondents was higher in our sample. In 2016, the mean age was 36 years among adults on community supervision in Hennepin County, with 35% aged 21-30 years (Hougham & Buskovick, 2018). However, we interviewed 36 respondents under 30, allowing us to describe participants’ experiences in this age range.

7 Although the categories are somewhat different, this breakdown in offense type is roughly comparable to the composition of the population of adults supervised in the county (Hougham & Buskovick, 2018).

8 At the time our interviews were conducted, medical marijuana was legal in Minnesota, but the process of getting approved was sufficiently cumbersome that none of our participants described being able to access legal dispensaries.

9 While the formal USDA evaluation criteria for food insecurity includes 18 questions, previous research has found that single-item questions are reasonable proxies for the longer scale. Food insecurity is more common among people who have been previously incarcerated and is associated with poorer future health (Testa & Jackson, 2019).
